# MsgaBpred: A B-cell epitope predictor integrating AlphaFold3-predicted structures with multi-scale GCNs and pre-trained language model ESM-C

**DOI:** 10.1371/journal.pcbi.1014195

**Published:** 2026-04-28

**Authors:** Shanyue Wang, Aoyun Geng, Zhenjie Luo, Yazi Li, Junlin Xu, Yajie Meng, Leyi Wei, Quan Zou, Zilong Zhang, Tao Wang, Feifei Cui

**Affiliations:** 1 School of Cyberspace Security (School of Cryptology), Hainan University, Haikou, China; 2 School of Computer Science and Technology, Hainan University, Haikou, China; 3 School of Mathematics and Statistics, Hainan University, Haikou, China; 4 School of Computer Science and Technology, Wuhan University of Science and Technology, Wuhan, Hubei, China; 5 School of Computer Science and Artificial Intelligence, Wuhan Textile University, Wuhan, Hubei, China; 6 Centre for Artificial Intelligence driven Drug Discovery, Faculty of Applied Science, Macao Polytechnic University, Macao SAR, China; 7 Institute of Fundamental and Frontier Sciences, University of Electronic Science and Technology of China, Chengdu, China; 8 Laboratory of Protein Structure and Function, Institute of Medicine and Pharmacy, Qiqihar Medical University, Qiqihar, Heilongjiang, China; Tel Aviv University, ISRAEL

## Abstract

Accurate prediction of B-cell epitopes plays a key role in facilitating advancements in vaccines, therapeutics, and diagnostics. In contrast to labor-intensive experimental approaches, computational strategies provide a more economical and efficient means of identifying potential epitopes. Existing methods are often limited by their reliance on experimentally resolved protein structures or by the use of lower-accuracy predicted structures. Sequence-based approaches, while fast, largely fail to capture the 3D spatial context essential for conformational epitopes. With the breakthroughs achieved by AlphaFold3 in predicting protein structures, we present **MsgaBpred,** the model to apply AlphaFold3-derived structures to B-cell epitope identification. Given only a protein sequence, our model employs a multi-scale graph convolutional network and additive attention to capture complex structural dependencies without relying on experimentally determined structures. The multi-scale design allows for effective modeling of both local and global contexts by aggregating information across different neighborhood ranges. Additionally, we leverage ESM-C, a more expressive protein language model than ESM-2, to enhance feature representation for B-cell epitope prediction. Extensive evaluations across multiple benchmark datasets demonstrate that MsgaBpred achieves competitive and robust performance; notably, it yields a statistically significant improvement in AUC compared to existing state-of-the-art methods. Moreover, the modular and scalable architecture of MsgaBpred holds promise for broader applications, including the structural analysis of other biomolecular entities such as nucleic acids and carbohydrates.

## Author summary

B cells arise from hematopoietic stem cells in the bone marrow and are essential mediators of humoral immunity, primarily through the secretion of antibodies that enable targeted and durable defense against invading pathogens. The precise segment of an antigen recognized and bound by an antibody is known as an epitope. Identifying B-cell epitopes accurately is vital for accelerating vaccine development, therapeutic antibody design, and diagnostic assays, yet experimental methods remain slow and expensive. While computational methods offer a faster route, they often fall short—either by needing high-resolution experimental structures or by ignoring the 3D conformation that defines most epitopes. To bridge this gap, we developed MsgaBpred, a new deep learning framework that starts with just a protein sequence and uses AlphaFold3 to generate reliable structural models. Our tool makes full use of the evolutionary and structural information to identify B-cell epitopes. Results show that our tool can identify B-cell epitopes more accurately and provide effective help for vaccine development.

## 1 Introduction

B cells originate from hematopoietic stem cells in the bone marrow [1] and play a central role in humoral immunity by producing antibodies that mediate specific and long-lasting responses against pathogens. The specific region on an antigen that binds to an antibody is referred to as an epitope or antigenic determinant [2]. B-cell epitopes are typically divided into two types: linear and conformational. Linear epitopes are composed of sequential amino acid residues, while conformational epitopes arise from non-contiguous residues that are brought into proximity through the protein’s three-dimensional folding. It is estimated that approximately 90% of B-cell epitopes are conformational, with only around 10% being linear [3]. Understanding B-cell epitopes is of great significance for vaccine design, disease diagnosis, and immunotherapy [4]. Traditional experimental methods for identifying B-cell epitopes include nuclear magnetic resonance (NMR) spectroscopy [5], X-ray crystallography [6], and cryo-electron microscopy (cryo-EM) [7]. Although these approaches yield precise structural information, they often require substantial time and computational resources. To address these challenges, numerous computational tools have been developed, offering efficient and cost-effective alternatives for epitope prediction.

Computational approaches for predicting B-cell epitopes are generally divided into sequence-based and structure-based methods. The former utilizes the antigen’s amino acid sequence as input data. Representative tools include BepiPred 2.0 [8], BepiPred 3.0 [9], CBTOPE [10], and SEPIa [11]. Among them, BepiPred 3.0 utilizes protein representations from the ESM-2 language model [12] and employs an early residue annotation strategy, achieving significant performance gains. Currently, BepiPred 3.0 is one of the best-performing sequence-based tools. However, these methods primarily excel at identifying linear epitopes and generally show limited effectiveness in recognizing conformational epitopes.

To overcome this challenge, structure-based methods leverage antigen structural information to more accurately identify conformational epitopes. For example, SEPPA 3.0 examines the influence of glycosylation on antigen surface regions and reveals a preference of antibodies for binding to glycosylated sites [13]. epitope3D employs graph-based features within a scalable machine learning framework [14]. GraphBepi constructs a protein graph based on AlphaFold2-predicted structures [15], which is then processed by an edge-enhanced deep neural network. DiscoTope-3.0 [16] uses reverse folding features derived from AlphaFold2-predicted [17] or experimentally resolved structures, trained via positive-negative label integration. WUREN, a multimodal model which combines sequence, graph, and structural features to predict epitope [18]. EpiGraph [19] leverages graph attention networks to model the spatial clustering characteristics of epitopes, incorporating structural and evolutionary information from pretrained models like ESM-2 [20] and ESM-IF1 [21].

Despite these advancements, structure-based models remain constrained by the limited availability of experimentally resolved structures. While AlphaFold2 has made it possible to predict protein structures from sequences, its accuracy still falls short of experimental methods, thereby limiting the reliability of downstream tasks such as epitope identification.

Recently, AlphaFold3 [22] has marked a significant breakthrough in protein structure prediction by substantially reducing dependence on deep Multiple Sequence Alignment (MSA) processing and leveraging diffusion models. It achieves unprecedented accuracy, outperforming previous computational methods by over 50% on benchmarks such as PoseBusters [23]. Recent studies have shown that alphafold3 has great potential in predicting B cell epitopes [24,25]. Concurrently, recent developments in protein language models have significantly improved the representation of evolutionary information [26–30]. The ESM family of models, particularly ESM-C, a parallel series to ESM-3 [31] generative models, focuses on learning latent biological representations and significantly outperforms ESM-2 under equivalent parameter settings. These advancements help overcome two persistent challenges in B-cell epitope prediction: the extraction of informative sequence features and the limited availability of high-quality structural data. In graph analysis and other domains, graph convolutional networks (GCNs) have been widely used [32–35]; however, conventional GCNs often struggle to model long-range dependencies and are mainly effective at capturing local interactions. A common workaround is to stack multiple GCN layers, but this may lead to over-smoothing, where node representations become indistinguishable. To overcome these limitations, we employ a two-layer multi-scale graph convolutional network that captures features across different receptive fields, effectively modeling both local and global structural dependencies. Additionally, residual connections are incorporated to mitigate over-smoothing effects.

In this study, we propose MsgaBpred, an end-to-end B-cell epitope prediction model that does not rely on experimentally determined native structures. Given only a protein sequence, the model uses AlphaFold3 to predict its 3D structure and integrates multiple advanced components, including ESM-C, ESM-IF1, a multi-scale graph convolutional network, and an additive self-attention mechanism. ESM-C and AlphaFold3 are used to extract sequence representations and structure information, respectively, while ESM-IF1 and DSSP [36] provide inverse folding and secondary structure features. These features are processed through the Multi-scale GCN and the Additive Attention module to capture both local structural patterns and global context. The final B-cell epitope prediction is performed by inputting the integrated low-dimensional features into a multilayer perceptron (MLP). Extensive evaluations on multiple independent benchmark datasets demonstrate that MsgaBpred outperforms existing state-of-the-art methods. This shows the strong generalization of MsgaBpred. Furthermore, we compared the structural information of proteins predicted by different structure prediction models with that of experimentally determined native protein structures. The results show that the structures predicted by AlphaFold3 exhibit only minor deviations from the native structures. An antigen from the test set was chosen as a representative case study, and the resulting analysis provides strong evidence of our model’s enhanced predictive capability relative to current approaches.

## 2 Materials and methods

### 2.1 Architecture of MsgaBpred

**Fig 1:**
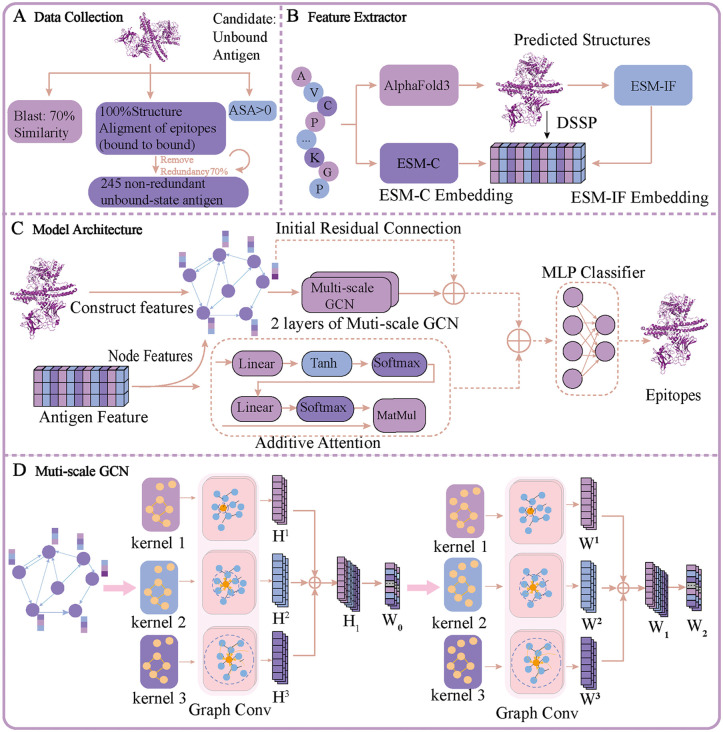
The overall framework of the proposed MsgaBpred model. **(A) Data Collection.** A total of 245 candidate unbound antigens were curated through rigorous filtering. Among them, 200 antigens were used for training and the remaining 45 for testing. **(B) Feature Extractor.** The antigen sequences are fed into the pretrained language model ESM-C to obtain evolutionary embeddings. Simultaneously, the sequences are submitted to AlphaFold3 to predict the 3D structures. These predicted structures are then processed through the inverse folding model ESM-IF1 and the DSSP algorithm to extract structural embeddings. Finally, the ESM-C embeddings, ESM-IF1 outputs, and DSSP-derived features are concatenated to form comprehensive antigen representations. All pretrained models are used as frozen feature extractors; only the additive attention, multi-scale GCN, and MLP modules are trained. **(C) Model Architecture.** The extracted features serve as inputs for two distinct modules: (1) an additive attention module, which captures global dependencies and key residue importance; and (2) a protein graph module where nodes correspond to amino acid residues, and edges represent spatial as well as sequential relationships inferred from the structure predicted by AlphaFold3. Node and edge features are processed by a two-layer multi-scale graph convolutional network (GCN). The outputs from both the attention module and the GCN are then concatenated and fed into a multi-layer perceptron (MLP) for final prediction. **(D) Multi-Scale GCN.** The multi-scale GCN consists of two stacked layers, each employing three parallel graph convolution kernels with receptive field sizes of 1, 2, and 3 hops to capture structural dependencies at multiple scales. The outputs from each kernel are fused via a linear transformation. This multi-scale design enhances the model’s ability to capture both short- and long-range interactions across the protein graph.

In this study, we propose a novel method, MsgaBpred, designed to accurately predict B-cell epitopes. As illustrated in **Fig 1B**, the antigen sequence is first processed through two pretrained models: ESM-C, which generates contextual sequence embeddings that capture evolutionary information, and AlphaFold3, which predicts the 3D structure of the protein. Structural features are then extracted from the predicted structure using two complementary methods: DSSP, which provides secondary structure, solvent accessibility, and torsion angle data; and ESM-IF1, which generates inverse folding embeddings. In parallel, the predicted protein structure is utilized to build a residue-level relational graph, with residues as nodes and spatial relationships encoded as edges.

On one branch of the model, the sequence embedding (from ESM-C), DSSP features, and inverse folding embeddings (from ESM-IF1) are fed into an additive attention module [37] to capture global contextual dependencies and identify key residues relevant to epitope formation. Simultaneously, as can be observed in Fig 1C, the three types of features are concatenated to form the node representations, while structural edge features are incorporated into a multi-scale graph convolutional network (GCN). This branch learns spatial and topological patterns that are indicative of structural epitope regions at different resolutions. The outputs from the additive attention module and the multi-scale GCN are subsequently concatenated and fed into a multilayer perceptron (MLP) to produce the final prediction of B-cell epitopes. This dual-branch architecture enables the model to effectively integrate sequence-level contextual cues and structure-aware relational patterns, thus enhancing prediction accuracy and robustness across diverse antigens.

### 2.2 Dataset

Our dataset is constructed following the rigorous curation protocol established in epitope3D [14], which enables large-scale annotation of conformational B-cell epitopes on unbound antigen structures. The process consists of two main stages: (i) epitope annotation on experimentally resolved antibody–antigen complexes, and (ii) transfer of these annotations to unbound antigen structures via sequence and structural alignment.

In the first stage, we retrieved all biological assemblies from the Protein Data Bank (PDB) with resolution ≤3 Å (as of May 2021). Antibody–antigen complexes were identified using the ANARCI tool [38], and antigen chains with at least 25 residues were retained. An antigen residue was labeled as an epitope if any of its heavy atoms lies within 4 Å of any antibody residue. This yielded 1,351 high-quality complexes, encompassing 40,842 epitope residues.

As illustrated in Fig 1A, in the second stage, we sought to propagate these epitope labels to unbound-state antigen structures—that is, PDB entries representing the same (or highly homologous) antigen protein in its free, uncomplexed form. These unbound structures are not extracted from the bound complexes; instead, they are independent experimental structures identified via blastp [39] search against the bound antigens, requiring ≥70% sequence identity. For each candidate unbound antigen, epitope labels from the corresponding bound antigen were transferred only if a high-fidelity structural alignment could be achieved between the two forms. Specifically, using the PyMOL [40] alignment module, we required that the epitope-containing segment aligns with perfect backbone correspondence—a condition referred to in the original epitope3D study as “100% structural alignment.” This ensures that only residues occupying equivalent 3D positions inherit the epitope label, preserving the conformational nature of the epitope.

After alignment and filtering, 343 unbound antigen structures were obtained. To minimize redundancy and prevent data leakage [41,42], we performed clustering at the cluster level based on sequence identity. Specifically, we used CD-HIT [43] with a 70% sequence identity threshold to group structurally similar antigens into clusters, thereby generating a non-redundant dataset of 245 antigen structures (comprising 168,739 surface residues, of which 3.56% are epitopes). This dataset is partitioned as follows: 200 structures for model training (used in 10-fold cross-validation), 45 structures for an independent external test set. Critically, no antigen in the test set shares ≥70% sequence identity with any antigen in the training set, effectively eliminating homology-based data leakage.

Class imbalance in training and test sets: In the training set (200 antigens), after applying our hybrid resampling strategy (random under-sampling and SMOTE), the final class distribution used for model training is 1:2 (epitope, non-epitope), i.e., 33.33% positive samples. In contrast, the independent external test set (45 antigens) retains its natural imbalance to reflect real-world conditions: epitope residues account for only 7.1% of all surface residues, corresponding to an imbalance ratio of approximately 1:13.

Furthermore, to better evaluate the generalizability of the model, we incorporated an external dataset proposed and curated by Høie MH [16], which comprises 24 antigens. These antigens are represented in the test set in three different forms: (i) experimentally resolved antigen structures, (ii) antigen structures predicted by AlphaFold2, and (iii) relaxation of the solved structures generated using FoldX [44].

### 2.3 Pre-trained large language models for evolving information

The recently developed protein language model ESM-C 6B (referred to as ESM-C) significantly outperforms the best-performing model in the ESM-2 [45–48] series. It is employed to extract sequence-based representations for individual protein residues. ESM-C is built upon the Transformer architecture and is trained in an unsupervised manner. Key architectural features include Pre-Layer Normalization (Pre-LN), rotary positional embeddings, and the SwiGLU activation function. Notably, no bias terms are used in either the linear layers or the layer normalization modules. Its ability to capture evolutionary information stems from the nature of its training data and objective, not merely its architecture. Specifically, ESM-C is trained on a massive, diverse corpus of protein sequences using a masked language modeling (MLM) objective: at training time, a subset of amino acid residues in each sequence is randomly masked, and the model is tasked with predicting the masked residues based on their surrounding context. Because protein sequences in this corpus represent evolutionarily related homologs, the model implicitly learns which amino acid substitutions are tolerated or co-vary across evolutionary time at each position.

At positions involved in structural or functional co-evolution, the model learns context-dependent dependencies, the identity of one residue influences the predicted distribution at another, even if they are distant in sequence. Crucially, unlike traditional methods, which explicitly compute conservation or co-variation from MSAs, ESM-C learns these patterns implicitly through deep contextual representation. The resulting residue-level embeddings thus encode rich evolutionary signals including conservation, co-evolution, and functional constraints. These embeddings provide evolutionarily informed priors for epitope prediction: epitope residues often exhibit distinct evolutionary signatures, which ESM-C can capture more effectively than non-evolutionary sequence features. This process enables ESM-C to internalize evolutionary constraint.

All in all, ESM-C captures evolutionary information not through explicit multiple sequence alignments, but by learning statistical patterns of amino acid co-occurrence and substitution across a vast corpus of homologous protein sequences during masked language model pretraining. This enables its embeddings to implicitly encode residue-level conservation, co-evolution, and functional constraints—key signals for distinguishing epitope from non-epitope regions. Through using ESM-C, we extracted contextualized residue-level embeddings, resulting in a 2,560-dimensional feature vector for each amino acid residue.

### 2.4 Protein structure prediction models

To comprehensively capture the spatial context of each residue within antibody–antigen complexes, we utilized AlphaFold3 to predict the tertiary structures of these protein complexes. AlphaFold3 enhances predictive accuracy by simplifying the original Evoformer module into a Pairformer architecture, which separately models pairwise and single-residue representations. Structure predictions using AlphaFold3 were performed via the AlphaFold Server (https://alphafoldserver.com). In addition to AlphaFold3, we also evaluated two related protein structure prediction models: AlphaFold2 and esmfold_v1(referred to as ESMFold [45]). AlphaFold2 relies heavily on MSA and attention mechanisms, offering higher structural accuracy than AlphaFold1 at the cost of slower prediction times. In contrast, ESMFold eliminates the need for MSAs, enabling faster inference while maintaining reasonable accuracy. The predicted structure of AlphaFold2 and ESMFold were conducted using ColabFold on Google Colab (https://github.com/sokrypton/ColabFold).

### 2.5 Inverse folding representations via ESM-IF1

To extract structure-aware features for each residue, we employed the esm_if1_gvp4_t16_142M_UR50 model (referred to as ESM-IF1) to generate inverse folding representations based on the structures predicted by AlphaFold3. For each residue, ESM-IF1 produces a 512-dimensional feature vector, which is incorporated as part of the protein’s structural representation. ESM-IF1 is a Transformer-based inverse folding model trained via self-supervised learning. It captures sequence evolutionary patterns constrained by structural context, thereby learning to represent the compatibility between sequences and their corresponding 3D conformations.

### 2.6 Secondary structure features using DSSP

We used the DSSP program to derive three different types of structural features from the protein structures predicted by AlphaFold3. These properties are as follows: Secondary Structure Profile: A 9-dimensional one-hot encoded vector${(SS}_{i})$representing the local secondary structure of each residue. The first eight dimensions represent the eight conventional secondary structure states, whereas the ninth dimension captures unknown or undefined secondary structures. Relative Solvent Accessibility (rASA): The absolute solvent accessible surface area (ASA) of each residue was normalized by the maximal ASA value of its respective amino acid type, resulting in a relative solvent accessibility score${(rASA}_{\text{i}}).$ Backbone Torsion Angles: The backbone dihedral angles *ϕ* (phi) and *ψ* (psi) were computed from atomic coordinates and transformed into a 4-dimensional feature vector using their sine and cosine $\left(\phi_{i}{,\psi }_{i}\right)$, ensuring rotational invariance and smoothness for learning algorithms. These three components were concatenated to form a 13-dimensional structural feature vector per residue, collectively referred to as the DSSP representation.

### 2.7 Additive attention module

To effectively extract global contextual information and identify key residues from the concatenated embeddings of ESM-C, DSSP, and ESM-IF1, we incorporate an additive attention mechanism based on a feedforward neural network. This mechanism enables the model to assign adaptive importance weights to different residues within the antigen sequence. Let $X\in R^{L*d}$denote the input matrix, where L is the sequence length and d is the embedding dimension of each residue. The additive attention score for the i-th residue is computed as follows:


$$\begin{array}{c} e_{i}=V^{T}tanh\left(Wx_{i}\right) \end{array}$$ (1)

where $x_{i}\in R^{d}$ is the embedding of the i-th residue, $W\in R^{d*d}$ is a learnable weight matrix, and $V\in R^{d}$ is a learnable vector that projects the nonlinearly transformed features to a scalar score.

The attention weights are then computed using a softmax function to ensure a normalized distribution over all residues:


$$\begin{array}{c} a_{i}=\frac{exp\left(e_{i}\right)}{\sum_{j=\text{1}}^{l}exp\left(e_{j}\right)} \end{array}$$ (2)

where $a_{i}$ denotes the attention weight corresponding to the i-th element, $e_{i}$ is the attention energy, and lis the sequence length.

The final sequence-level representation $Z\in R^{d}$ is obtained by a weighted sum of all residue embeddings:


$$\begin{array}{c} Z=\sum_{i=\text{1}}^{L}a_{i}x_{i} \end{array}$$ (3)

This attention mechanism allows the model to adaptively focus on the most informative residues within the sequence, which is particularly beneficial for B-cell epitope prediction, where only a subset of residues contribute to antigenicity. Moreover, the attention distribution provides interpretability, offering insights into the spatial and functional organization of epitope regions.

### 2.8 Protein graph construction

To model the structural dependencies among amino acid residues within a protein, we represent each protein as a graph G = (N, E, R), following the approach described in [49]. Here, $N=\left\{n_{\text{1}},n_{\text{2}},{\ldots ,n}_{N}\right\}$ denotes the set of nodes, where each node corresponds to a residue in the protein. E is the set of edges, and R denotes the set of edge types. The connectivity between nodes is determined based on both 3D structural information and sequence proximity, aiming to capture both local sequential dependencies and long-range spatial interactions within the protein. During the graph construction process, we define the following three types of edges:

1. Sequential Edges: An edge is added between two residues i and j if their positions in the primary sequence satisfy $|i-j|<d_{seq}$. These edges capture local connectivity along the sequence.
2. Radius Edges: An edge is added between residues i and j if the Euclidean distance between their Cα atoms satisfies $|{|c}_{i}-c_{j}|{|}_{\text{2}}<d_{r}$ and their sequence separation satisfies $|i-j|\geq d_{long}$. These edges model non-local spatial interactions that arise due to the protein’s folded structure.
3. K-Nearest Neighbor (KNN) Edges: For each residue, we identify its K nearest neighbors in 3D space based on Euclidean distance, and add edges to those neighbors if $|i-j|\geq d_{long}$. This strategy improves graph connectivity and enables the model to learn long-range dependencies between spatially close but sequentially distant residues.

where $d_{seq}$is the maximum sequence separation allowed for sequential edges. Specifically, an edge is added between residues i and j if and only if $\text{0}<|i-j|<d_{seq}$, $d_{r}$ is the spatial distance threshold used for constructing spatial adjacency edges. And $d_{long}$ means minimum sequence distance required to establish long-range edges (i.e., Radius edges and KNN edges). We only add these edges if $|i-j|\geq d_{long}$. To avoid ambiguity, we enforce $d_{long}>d_{seq}$, creating a gap between the two regimes. Residues with sequence separation in the interval $d_{seq}<|i-j|<d_{long}$ do not receive sequential edges, and spatial edges are only added if they also satisfy the 3D distance or KNN criteria. This design prevents redundancy: short-range spatial proximity is already implicitly modeled via sequential edges and local GCN propagation, while true long-range contacts are explicitly encoded via spatial edges. We set the sequential distance threshold $d_{seq}=\text{3}$, $d_{r}=\text{1}\text{0}$, the number of neighbors k = 10 and $d_{long}=\text{5}$, following the set in [49].

This multi-edge construction scheme ensures that the graph representation preserves both the topological layout of the sequence and the biophysical constraints of the folded structure, enabling the downstream graph neural network to learn both local and global structural patterns relevant to epitope recognition.

### 2.9 Node feature

Since the original feature dimensions of ESM-C embeddings, ESM-IF1 inverse folding representations, and DSSP features differ significantly, directly concatenating them may lead to excessive noise or feature imbalance. Each feature modality is projected independently into a 256-dimensional latent space using modality-specific linear layers. This prevents high-dimensional embeddings from overwhelming lower-dimensional but biologically informative features such as those from DSSP. Specific steps are as follows, each feature type is first projected into a unified latent space via linear transformation and layer normalization before concatenation. Taking DSSP features as an example, let the initial DSSP representation be $X_{{DSSP}_{\text{0}}}\in R^{\text{1}\text{3}*N}$, where N is the number of residues. It is first linearly projected to a 256-dimensional space:


$$\begin{array}{c} X_{{DSSP}_{\text{1}}}=\left(W_{\text{0}}X_{{DSSP}_{\text{0}}}+b_{\text{0}}\right),W_{\text{0}}\in R^{\text{2}\text{5}\text{6}*\text{1}\text{3}} \end{array}$$ (4)

This is followed by a layer normalization and ReLU activation:


$$\begin{array}{c} X_{{DSSP}_{\text{2}}}=Relu\left(\frac{X_{{DSSP}_{\text{1}}}-\mu_{{DSSP}_{\text{1}}}}{\sigma_{{DSSP}_{\text{1}}}}\cdot \gamma +\beta \right) \end{array}$$ (5)

where $\mu_{{DSSP}_{\text{1}}}$ and $\sigma_{{DSSP}_{\text{1}}}$ denote the mean and standard deviation of $X_{{DSSP}_{\text{1}}}$, and γ and β, are learnable scale and shift parameters.

Similar transformations are applied to the ESM-C and ESM-IF1 embeddings to bring them to the same 256-dimensional space. The final **node feature matrix** is obtained by concatenating all three normalized and transformed representations:


$$\begin{array}{c} X_{\text{0}}=Concat\left(X_{ESM-CEmbedding},X_{ESM-IF\text{1}Embedding},X_{DSSP}\right)\in R^{N*\text{7}\text{6}\text{8}} \end{array}$$ (6)

The resulting node features integrate multi-source information, including evolutionary signals, geometric and physicochemical structure features, and structure-conditioned sequence constraints, offering a rich and comprehensive representation for downstream epitope prediction. Although mapping DSSP to a high-dimensional latent space may introduce noise, experiments have shown that the model performs better when mapping it to a high-dimensional space compared to keeping the DSSP dimensions unchanged. As shown in S5 Table, we tried reducing only the high-dimensional features while keeping DSSP at its original dimensionality, the result shows that compared to keeping the DSSP dimensions unchanged, mapping DSSP to a high-dimensional representation resulted in a 1.3% increase in the model’s AUPR and a 1.2% increase in the F1 score.

### 2.10 Multi-scale graph convolutional network module

To effectively capture the structural characteristics of proteins, we design a multi-scale graph convolutional network (GCN) that incorporates multiple receptive field sizes within each layer [50]. By incorporating this design, the model is enabled to learn residue interactions at both local and distant scales, addressing the inherent limitations of conventional GCNs with fixed, restricted receptive fields.

As illustrated in Fig 1D, our model stacks two GCN layers, each of which performs parallel convolutions with varying neighborhood sizes, thereby alleviating the over-smoothing problem commonly seen in deep GCNs. Additionally, residual connections are introduced to preserve low-level features and further mitigate over-smoothing. Let the input node features be $X_{\text{0}}\in R^{{N*d}_{\text{0}}}$, where N is the number of residues (nodes) and $d_{\text{0}}$ is the dimensionality of the concatenated input features. These features are first projected into a lower-dimensional hidden space of dimension $d_{\text{1}}$ to facilitate efficient message passing in subsequent graph.

For each GCN layer, we perform graph convolutions with multiple kernel sizes k∈{1,2,3}, corresponding to different orders of neighborhood aggregation.

The update for node i at layer 1 with kernel size k is given by:


$$\begin{array}{c} \overset{\left(\text{1},k\right)}{\underset{i}{H}}=\sigma \left(\sum_{j\in N_{k}(i)}^{N}\frac{\text{1}}{\sqrt{deg(i)deg(j)}}\cdot \overset{\text{1}}{\underset{k}{W}}\cdot \overset{\text{1}}{\underset{j}{h}}\right),k\in \left\{\text{1},\text{2},\text{3}\right\} \end{array}$$ (7)

where $N_{k}(i)$ denotes the k-hop neighborhood of node i, deg(i) is the degree of node i, $\overset{\text{1}}{\underset{k}{W}}$ is the learnable weight matrix for kernel k at layer 1, σ is a nonlinear activation function (ReLU), $\overset{\left(\text{1},k\right)}{\underset{i}{H}}$ is the feature vector of node i at layer 1. Here, $\overset{\text{1}}{\underset{j}{h}}$ denotes the initial feature vector of node *j* (i.e., the concatenated and normalized representation from ESM-C, ESM-IF1, and DSSP.

After computing the outputs for each kernel size, the results are concatenated and passed through a linear transformation for feature fusion:


$$\begin{array}{c} H_{\text{1}}=Concat\left(\overset{\left(l\right)}{\underset{\text{1}}{H}},\overset{\left(l\right)}{\underset{\text{2}}{H}},\overset{\left(l\right)}{\underset{\text{3}}{H}}\right),H^{\left(l\right)}\in R^{\text{3}*d_{\text{1}}} \end{array}$$ (8)

$$\begin{array}{c} W_{\text{0}}=Relu\left(H^{\left(l\right)}W_{\text{1}}+b_{\text{1}}\right) \end{array}$$ (9)

The output of layer l is then fed into the next layer, and the process is repeated. To preserve low-level information and prevent feature homogenization across nodes, we introduce a residual connection between the initial and final layer outputs:


$$\begin{array}{c} H=\lambda H^{\text{0}}+(\text{1}-\lambda )W_{\text{2}} \end{array}$$ (10)

Where λ∈[0,1] is a learnable scalar that balances the contribution of low- and high-level representations. This multi-scale, residual-enhanced GCN architecture enables the model to capture rich spatial hierarchies and structural contexts essential for precise B-cell epitope prediction.

### 2.11 Model training

To enhance B-cell epitope identification, we utilized the Binary Cross-Entropy (BCE) loss function, a widely used choice for binary classification problems. BCE loss quantifies the difference between predicted probabilities and true binary labels, encouraging the model to assign higher probabilities to positive instances and lower probabilities to negative ones. The BCE loss function is defined as:


$$\begin{array}{c} L_{BCE}=-\frac{\text{1}}{N}\left[y_{i}log\left(p_{i}\right)+(\text{1}-y_{i})log(\text{1}-p_{i})\right] \end{array}$$ (11)

where N is the number of samples, $y_{i}\in \left\{\text{0},\text{1}\right\}$ is the ground truth label for the $i^{th}$ sample,$p_{i}\in \left\{\text{0},\text{1}\right\}$ is the predicted probability for the positive class. Adam optimizer was used to optimize the BCE Loss function.

### 2.12 Evaluation metrics

To comprehensively evaluate the performance of our model, we employed seven widely used metrics [30,51], including precision (Pre), recall (Rec), F1-score (F1), Matthews correlation coefficient (MCC), area under the precision-recall curve (AUPR), area under the receiver operating characteristic curve (AUC), and balanced accuracy score (BACC). These metrics assess both threshold-dependent and threshold-independent aspects of binary classification performance [52–55]. The definitions and formulas are as follows:


$$\begin{array}{c} Pre=\frac{TP}{TP+FP} \end{array}$$ (12)

$$\begin{array}{c} Rec=\frac{TP}{TP+FN} \end{array}$$ (13)

$$\begin{array}{c} F\text{1}=\text{2}*\frac{Precision*Recall}{Precision+Recall} \end{array}$$ (14)

$$\begin{array}{c} MCC=\frac{TP*TN-FN*FP}{\sqrt{\left(TP+FP)*(TP+FN)*(TN+FP)*(TN*FN\right)}} \end{array}$$ (15)

$$\begin{array}{c} BACC=\frac{\text{1}}{\text{2}}\left(\frac{TP}{TP+FN}+\frac{TN}{TN+FP}\right) \end{array}$$ (16)

where:

TP: True Positives — correctly predicted epitope (binding) residues,

TN: True Negatives — correctly predicted non-epitope (non-binding) residues,

FP: False Positives — non-epitope residues incorrectly predicted as epitopes,

FN: False Negatives — epitope residues incorrectly predicted as non-epitopes.

Threshold-based metrics (Pre, Rec, F1, MCC, BACC) are calculated using a classification threshold that is determined by maximizing the F1-score on the validation set. In contrast, AUC and AUPR are threshold-independent metrics that reflect the model’s ranking capability across the entire prediction score range. Overall, these metrics enable a thorough and balanced assessment of the model’s performance in B-cell epitope identification, accounting for the intrinsic class imbalance within epitope datasets.

Residue-level metrics were primarily employed because B-cell epitopes are defined by specific biochemical interactions at the residue-antibody interface. This high-resolution approach is the established standard in epitope prediction benchmarking, enabling MsgaBpred to be directly compared with other state-of-the-art predictors.

The proposed model was assessed using 10-fold cross-validation on 200 antigens from the Epitope3D training set, with evaluation performed on an independent Epitope3D test set. Upon completion of training for each fold, the resulting model was directly evaluated on the test set, and the area under the receiver operating characteristic curve (AUC) was measured. The model with the highest AUC across the ten folds was selected as the final (best-performing) model and used for downstream analysis. In addition, we computed the average performance across all ten folds. Given the class imbalance in the dataset, a smaller number of epitope residues compared to non-epitope residues, we emphasized AUPR, MCC, and BACC during evaluation. These metrics are known to provide robust and reliable assessments of model performance under imbalanced conditions, ensuring that both sensitivity and specificity are adequately captured.

## 3 Result and discussion

### 3.1 Performance comparison of feature combinations

**Fig 2:**
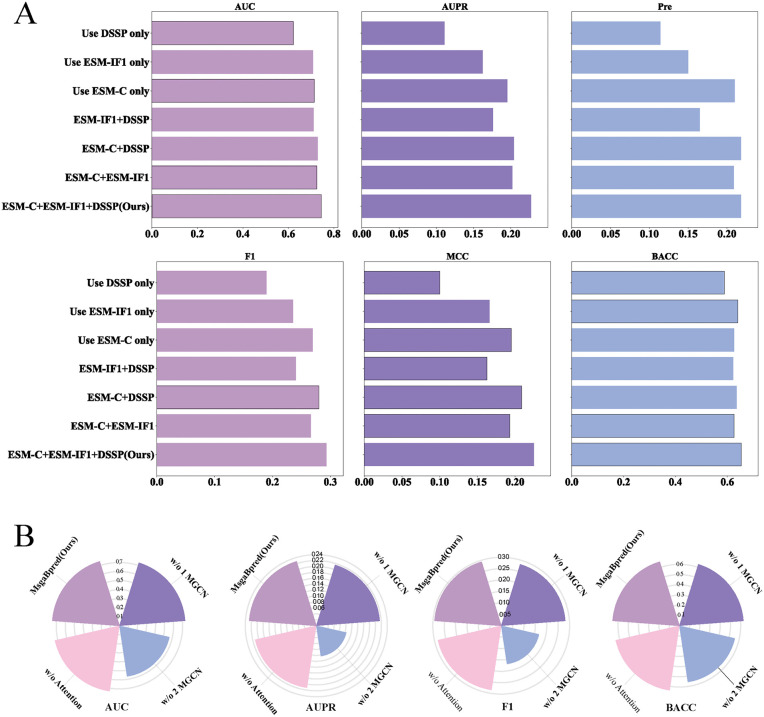
A. The performance of using different feature combinations on the independent test data. The evaluation metrics include AUC, AUPR, Precision, F1-score, MCC, and BACC. **B**. w/o means without the corresponding module. Ablation study results showing the performance of the full model and three variant architectures across five metrics: AUC, AUPR, Precision, F1-score, and MCC.

To evaluate the impact of each input feature, we performed ablation studies by using each feature type individually: ESM-C, ESM-IF1, or DSSP. As illustrated in S1 Table and Fig 2A, the model using only ESM-C embeddings outperformed the other two: its AUC and AUPR scores were 0.6% and 3.3% higher than those obtained using ESM-IF1, and 9.2% and 8.4% higher than those using DSSP features alone. It was observed that sequence embeddings derived from the pretrained language model ESM-C provide richer information for B-cell epitope prediction compared to inverse folding embeddings from ESM-IF1 and secondary structure features generated by DSSP.

In addition, we evaluated the effectiveness of feature combinations by conducting feature exclusion experiments, where one feature component was removed at a time from the full feature set. As illustrated in Fig 2A, removing any one of ESM-C, ESM-IF1, or DSSP led to performance degradation in both AUC and AUPR, confirming the complementary contributions of these features. Notably, excluding ESM-C resulted in the most significant drop—3.3% in AUC and 5.1% in AUPR—further validating that ESM-C provides the most critical information among the three. The superior performance of ESM-C embeddings may be attributed to the advanced architecture and larger training corpus used during its pretraining, enabling it to capture more comprehensive evolutionary and contextual information at the residue level.

### 3.2 Model performance comparison on the Epitope3D Dataset

To validate the efficacy of the proposed method, MsgaBpred, we performed a comprehensive benchmark evaluation using the widely recognized Epitope3D dataset. Following established protocols, we restricted the evaluation to surface-exposed residues, which were defined using a relative solvent accessibility (RSA) threshold of 0.15 [56], ensuring consistency with prior studies.

**Fig 3:**
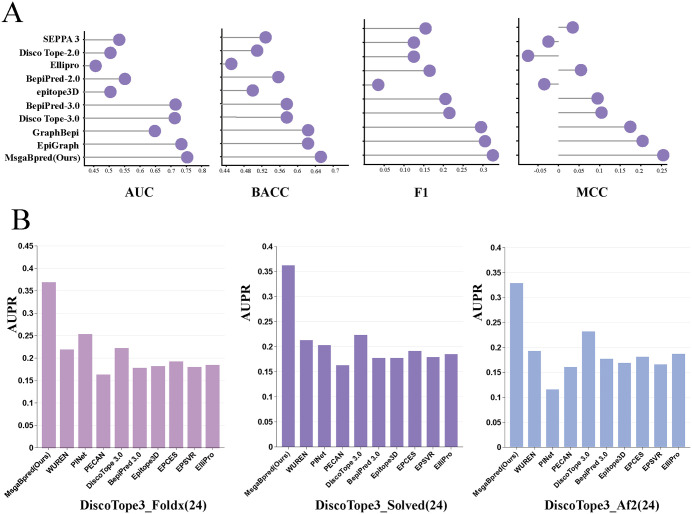
A. Performance of MsgaBpred compared to state-of-the-art approaches on the Epitope3D benchmark. **B.** Comparison of MsgaBpred performance with state-of-the-art methods across three distinct test datasets, DiscoTope3_Foldx, DiscoTope3_Solved, and DiscoTope3_Af2. The blue bar graph represented by MsgaBpred is the highest in all three graphs, while the heights of the bar graphs of other colors are much smaller than it.

We tested MsgaBpred alongside a comprehensive suite of competitive models, including SEPPA 3 [13], ElliPro [57], DiscoTope-2.0 [58],BepiPred-2.0 [8], epitope3D [14], BepiPred-3.0 [9], DiscoTope-3.0 [16], GraphBepi [32], and EpiGraph [19]. All models were evaluated on the same held-out test set of 45 antigens, using the same dataset to train, to ensure a fair and reproducible comparison. The results distinctly demonstrate that MsgaBpred outperforms other methods. As illustrated in Fig 3A and S2 Table, compared to the strongest baseline method, EpiGraph, our model achieved noticeable improvements across several key metrics. Specifically, MsgaBpred achieved higher scores in AUC, BACC, F1-score, and MCC, with respective gains of approximately 2%, 3%, 2%, and 5%. These consistent advantages across multiple evaluation criteria underscore the robustness of our model’s predictions. The result of MsgaBpred is further visualized through a radar plot, where it consistently occupies the outermost layer, reflecting comprehensive and balanced improvements over existing methods. Furthermore, the noticeable margin between MsgaBpred and the second-best model further affirms the effectiveness of incorporating both multi-scale structural information and sequence-based contextual features into the prediction framework. Overall, the benchmark results convincingly show that MsgaBpred sets a new state-of-the-art in B-cell epitope prediction, especially in accurately identifying conformational epitopes, which are essential for antibody binding and vaccine design.

### 3.3 Statistical evaluation of model performance

Although our model demonstrates better performance across multiple evaluation metrics compared to existing baseline models, such a claim cannot be substantiated without rigorous statistical validation. To this end, we conducted a comprehensive statistical comparison between our model and EpiGraph (the strongest-performing baseline) on the Epitope3D test dataset.

Specifically, we employed the DeLong test to assess whether the difference in AUC between the two models is statistically significant. The DeLong test is a widely accepted non-parametric method for comparing AUCs of two correlated ROC curves. For threshold-dependent metrics including BACC, MCC, and F1 score, we applied stratified bootstrap resampling to estimate both p-values and confidence intervals. In this framework, the p-value quantifies the probability of observing the measured performance difference under the null hypothesis that the two models perform identically.

As shown in the Table 1, the DeLong test for AUC yielded a p-value of 0.0212 (< 0.05), indicating that the observed AUC improvement of our model (0.7287) over EpiGraph (0.7103) is statistically significant. This suggests that, under the null hypothesis of no true difference in discriminative ability, the likelihood of observing such a difference purely by chance is only 2.12%. Therefore, we conclude that our model exhibits a statistically significant enhancement in global discriminative capacity.

**Table 1:** The result of model performance with statistical analysis. Delta means the difference between MsgaBpred and EpiGraoh.

Metric	Delta	P-values	Confidence
AUC	0.0185	0.0212	[-0.0346, 0.0346]
BACC	-0.0003	0.974	[-0.0192, 0.0186]
MCC	-0.0060	0.696	[-0.035, 0.0212]
F1	-0.0048	0.705	[-0.0294, 0.0183]

In contrast, for threshold-dependent metrics (BACC, F1, and MCC), no statistically significant differences were observed (all P-values > 0.05). This finding is not inconsistent with the AUC result, as these metrics capture fundamentally different aspects of model performance. AUC is a threshold-independent measure that reflects the model’s overall ability to rank positive instances higher than negative ones across all possible classification thresholds. Conversely, BACC, F1, and MCC are evaluated at a fixed decision threshold and thus represent localized performance under a specific operating point. Moreover, the absolute differences in these metrics are minimal. For instance, the BACC difference is merely 0.0003 and the F1 difference is only 0.0048—substantially smaller than the AUC gap of 0.0185.

In summary, while the absolute gains in threshold-dependent metrics over the baseline EpiGraph are marginal and not statistically significant, the improvement in AUC is both meaningful and statistically validated. This provides robust evidence that our model achieves a genuine enhancement in overall performance.

### 3.4 Model component evaluation

While the architecture of MsgaBpred is a key contributor to its performance, the choice of pretrained models for sequence and structure representation also plays a critical role. To validate our design decisions, we conducted additional ablation studies by substituting core components with alternative state-of-the-art models.

Although ESM-C is reported to outperform ESM-2 in general protein representation tasks [31], its advantage in the specific context of B-cell epitope prediction had not been empirically demonstrated. To address this, we replaced ESM-C with two widely used alternatives: ESM-2 (150M), ESM-2 (650M) [45] and ProtT5-XL-UniRef50 [59], while keeping all other components including AlphaFold3 structures, ESM-IF1, DSSP, GCN, and attention unchanged. As shown in Table 2, MsgaBpred with ESM-C achieves the highest performance across all metrics. Specifically, it yields an AUC of 0.744, compared to 0.731 for ESM-2(650M) and 0.728 for ProtT5 with a relative improvement of 1.3% and 2.6% in AUC, respectively, confirming that ESM-C provides more efficient features for identifying epitope residues.

**Table 2:** The performance of using different sequence representations on the Epitope3D dataset.

Model	AUC	AUPR	Pre	F1	MCC	BACC
ProtT5	0.728	0.195	0.196	0.263	0.190	0.631
ESM-2(150M)	0.727	0.185	0.186	0.256	0.184	0.633
ESM-2(650M)	0.731	0.188	0.187	0.260	0.186	0.632
ESMC(Ours)	**0.744**	**0.227**	**0.218**	**0.293**	**0.225**	**0.654**

We also explored alternative structure-aware representation strategies recently proposed in the literature. Specifically, we evaluated 3Di tokens [60], a discrete structural alphabet derived from protein backbone conformations as a compact encoding of local structural context. As shown in Table 2, when integrated into MsgaBpred (replacing ESM-IF1), the 3Di tokens achieved an AUC of 0.725 and AUPR of 0.211 on the Epitope3D test set, underperforming our default selection model ESM-IF1. We further tested SaProt [61], a unified structure-aware language model that jointly embeds sequences and 3D coordinates. As shown in Table 3, replacing ESM-IF1 with SaProt’s single embedding yielded the AUC of 0.741 and AUPR of 0.223. Although it is competitive but still inferior to our modular fusion approach.

**Table 3:** The performance of using different structure representations on the Epitope3D dataset.

Model	AUC	AUPR	Pre	F1	MCC	BACC
3Di tokens	0.725	0.211	0.218	0.280	0.210	0.638
Saprot	0.741	0.223	**0.221**	0.291	0.224	0.651
ESM-IF1(Ours)	**0.744**	**0.227**	0.218	**0.293**	**0.225**	**0.654**

These results, confirm that for conformational B-cell epitope prediction, combining a powerful sequence encoder (ESM-C) with complementary, interpretable structural descriptors (ESM-IF1 + DSSP) yields more discriminative representations than other model combinations.

### 3.5 Module ablation study

To assess the contribution of different architectural components to overall model performance, we performed a series of module ablation studies. Specifically, we compared the full model against three variant models: (1) a model combining additive attention with a single-layer multi-scale GCN. (2) a model with only the additive attention module, and (3) a model with only a two-layer multi-scale graph convolutional network (GCN)

As illustrated in Fig 2B and S3 Table, removing the additive attention module resulted in a 1.1% drop in AUC and a 1.5% drop in AUPR, indicating that additive attention effectively captures global dependencies among residues and adaptively emphasizes important positions in the sequence. Reducing the number of GCN layers from two to one led to a 1.4% decrease in AUC and a 1.2% decrease in AUPR, suggesting that stacking GCN layers mitigates the inherent limitation of GCNs in modeling long-range interactions. The most significant performance degradation was observed when the entire multi-scale GCN module was removed, with AUC and AUPR dropping by 17.6% and 12.3%, respectively. These findings emphasize the essential role of the GCN module, particularly in accurately modeling conformational B-cell epitopes that depend strongly on three-dimensional structural context. In summary, the combination of multi-scale GCN and additive attention improves the model’s accuracy in predicting conformational B-cell epitopes, demonstrating the value of integrating both structural and sequence-level contextual information.

### 3.6 Model generalization to external datasets

The generalization performance of the proposed model was evaluated by comparing MsgaBpred with nine established tools—WUREN [18], PINet [62], PECAN [63], DiscoTope 3.0 [16], BepiPred 3.0 [9], epitope3D [14], EPCES [64], EPSVR [64], and ElliPro [57]—on three antigen–antibody complex datasets: DiscoTope3_Foldx, DiscoTope3_Solved, and DiscoTope3_Af2. The sequence similarity between the external test set and the training set is 0.5254%, and that between the external test set and the internal test set is 0.5176%, indicating negligible overlap. For comparison, we used each method’s official web server to generate predictions on the same test sets. As illustrated in Fig 3B and S4 Table, MsgaBpred consistently outperforms all other models across all three datasets. The model does not just excel on a single dataset—it maintains top performance on diverse structural contexts, further highlighting its robustness. Additionally, we observed that MsgaBpred achieved a slightly higher AUPR score on the DiscoTope3_Foldx dataset compared to DiscoTope3_Solved, with a 0.2% increase, and that DiscoTope3_Solved outperformed DiscoTope3_Af2 by 3.3% in AUPR. This indicates that our model performs better on energy-minimized structures (Foldx) and experimentally solved structures than on structures predicted by AlphaFold2. These findings suggest that protein structure quality does influence the prediction accuracy of MsgaBpred, with optimized or experimentally determined structures yielding better results. Nonetheless, even on predicted or lower-quality structures, MsgaBpred still surpasses other models, confirming its robust and reliable predictive ability.

### 3.7 Impact of predicted structure quality on model performance

Given that our model relies on protein structures predicted by AlphaFold3, it is essential to investigate how the quality of predicted structures affects the model’s ability to identify B-cell epitopes. Furthermore, we further evaluated the model using protein structures generated by ESMFold and AlphaFold2, in addition to experimentally determined native structures as a reference standard.

**Fig 4:**
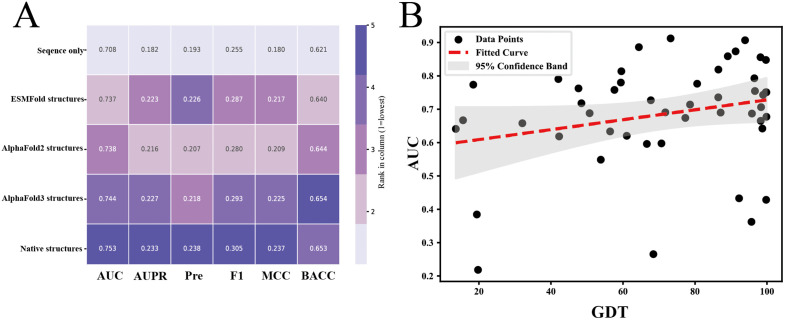
A. B-cell epitope prediction performance of MsgaBpred using different sources of protein structural information. A heatmap is used to visualize performance across evaluation metrics, with color intensity corresponding to the ranking of each model—darker colors indicate better performance. B. A positive association was identified between the structural quality predicted by AlphaFold3 (as measured by GDT) and the performance of MsgaBpred on the Epitope3D test set. Black scatter points indicate the GDT and AUC values for each individual protein, while the red line illustrates the functional relationship between GDT and AUC within the graph.

As shown in the Fig 4A, models using native structures yielded the best predictive performance, with the deepest color indicating the highest scores. However, models built on AlphaFold3-predicted structures demonstrated nearly comparable performance, with only a 0.9% drop in AUC and a 0.6% drop in AUPR compared to those using native structures. This finding is particularly encouraging—it suggests that high-quality predicted structures can effectively replace experimental structures in B-cell epitope prediction tasks, thereby significantly reducing both time and cost associated with experimental protein structure determination. In contrast, structures predicted by AlphaFold2 and ESMFold resulted in lower prediction performance, highlighting the superiority of AlphaFold3 in capturing structural features relevant to epitope recognition. On the other hand, when the model was trained and tested using only sequence-based inputs, its predictive performance declined substantially. This clearly demonstrates the critical role of structural information in accurately identifying conformational B-cell epitopes.

To further quantify the impact of structure quality, we calculated the Global Distance Test (GDT) [65] scores between AlphaFold3-predicted structures and their corresponding native structures using the LGA web server. As illustrated in Fig 4B and S1 Fig, each black dot represents a test case. The data show a positive correlation between GDT scores and AUC values, indicating that higher structural similarity to native proteins leads to better predictive performance. The relationship between AUPR and GDT is provided in S1 Fig. These results confirm that the accuracy of predicted protein structures has a direct and positive effect on the model’s ability to identify B-cell epitopes.

### 3.8 Case study

**Fig 5:**
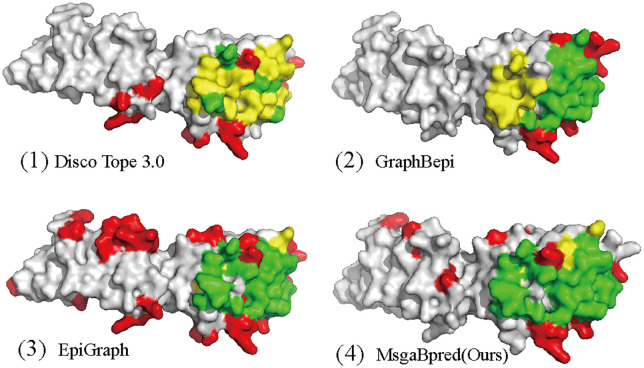
Visualization results of MsgaBpred, EpiGraph, GraphBepi, and Disco Tope3.0 on test data (PDB ID: 3BIK, Chain: A). True positives, false negatives, and false positives are colored in green, red, and yellow, respectively.

To provide a clear illustration of our model’s predictive power, we visualized the predicted results using PyMOL [40]. **Fig 5** presents the comparative visualization results for four models—DiscoTope 3.0 [16], GraphBepi [15], and EpiGraph [19] MsgaBpred —on a representative test sample (PDB ID: 3BIK, Chain: A). The 3BIK_A sample contains 216 residues, among which 27 are annotated as B-cell epitopes. Detailed performance metrics for each model are summarized in **Table 4**. MsgaBred correctly predicted 23 true positives (TP), with 17 false positives (FP) and 4 false negatives (FN). It achieved an AUPR of 0.5087, AUC of 0.88, and an F1-score of 0.6866. EpiGraph identified 18 TPs, 18 FPs, and 9 FNs, resulting in an AUPR of 0.3758, AUC of 0.7847, and F1-score of 0.5055. GraphBepi achieved 23 TPs, but with a higher 37 FPs and 4 FNs, giving an AUPR of 0.3454, AUC of 0.8259, and F1-score of 0.5287. DiscoTope 3.0 had significantly lower performance, predicting only 5 TPs, with 14 FPs and 22 FNs, resulting in an AUPR of 0.1525, AUC of 0.5548, and F1-score of 0.2174. These visual and quantitative comparisons further demonstrate the effectiveness of MsgaBpred in B-cell epitope prediction on real structural samples.

**Table 4:** The predictive performance on the example (PDB ID:3BIK, Chain:A) of different method.

Methods	TP	FP	FN	F1	AUPR	AUC	MCC
DiscoTope 3.0	5	14	22	0.2174	0.1525	0.5548	0.1278
GraphBepi	23	37	4	0.5287	0.3454	0.8259	0.4824
EpiGraph	18	18	9	0.5714	0.3758	0.7847	0.5055
MsgaBred(Ours)	23	17	4	0.6866	0.5087	0.88	0.6475

### 3.9 Biological interpretability

To elucidate the biological features captured by MsgaBpred, we analyzed its predictive logic at both residue and patch levels. At the residue level, the model leverages contextualized embeddings from ESM-C to internalize implicit evolutionary constraints, such as conservation and co-evolutionary patterns, which are key signals for distinguishing epitopes from non-epitope regions. Furthermore, the integration of inverse folding representations via ESM-IF1 allows the model to assess the compatibility between the protein sequence and its 3D conformation, ensuring that predicted residues are structurally stable and accessible.

**Fig 6:**
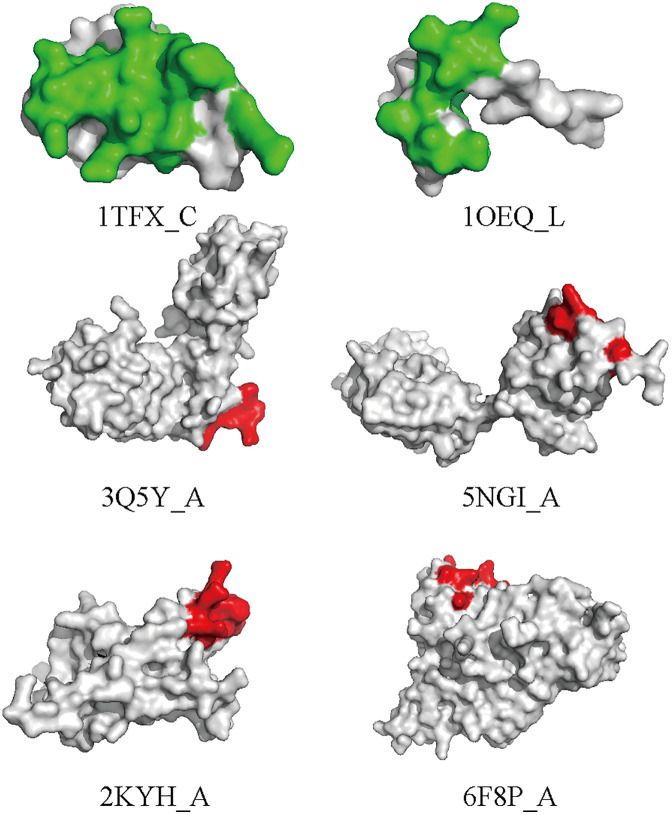
Visualization results of MsgaBpredon test data (PDB ID: 1TFX, Chain: C; PDB ID: 1OQE, Chain: L). True positives are colored in green. Residues are colored in red.

At the patch level, MsgaBpred moves beyond linear sequence limitations by employing a multi-scale GCN architecture. By aggregating information across 1, 2, and 3-hop neighborhoods, the model captures the “spatial clustering” property inherent to B-cell epitopes. This mechanism enables the model to bridge residues that are sequentially distant but spatially adjacent, effectively identifying them as an integrated 3D surface patch. As shown in **Fig 6**, in the case study of 1OQE_L and 1TFX_C, while the true positive residues are scattered across the primary sequence, MsgaBpred successfully identifies them as a cohesive structural cluster. This transition from residue-level identification to patch-level recognition demonstrates the model’s ability to capture the functional topography of antibody-antigen interfaces, providing actionable insights for vaccine and therapeutic design.

For antigens with high structural complexity like 3BIK_A, MsgaBpred successfully identifies anchor residues that function as structural hubs. These residues maintain a high density of spatial contacts with their neighbors, providing a stable platform for signal propagation. By utilizing the Multi-scale GCN, the model aggregates features from these hubs to form a cohesive 3D surface patch. Biologically, these high-connectivity regions represent stable conformational epitopes where multiple residues are brought together by tertiary folding, creating an ideal binding interface for antibody paratopes.

Conversely, we analyzed antigens where the model showed limited sensitivity. As illustrated in **Fig 6**, a common feature among these failure cases is topological isolation. In these instances, the true epitope residues are located on highly extended, flexible loops or distal structural elements with very low spatial connectivity.Because these residues lack sufficient topological support from the surrounding environment, the Multi-scale GCN cannot aggregate a rich structural context from the 1–3 hop neighborhood. Consequently, the immunogenic signal is diluted by background structural noise, leading to false negatives. This systematic examination clarifies that while MsgaBpred is exceptionally robust for spatially supported conformational patches, its performance is inherently bounded by the antigen’s local structural density.

## 4 Conclusion

Accurate identification of B-cell epitopes is essential for the development of effective vaccines, therapeutics, and diagnostic tools. In this study, we introduce **MsgaBpred**, the first known model to integrate AlphaFold3-predicted protein structures with multi-scale graph convolutional networks and an additive attention mechanism for B-cell epitope prediction. Unlike traditional structure-based models that rely on experimentally resolved structures, MsgaBpred requires only protein sequences as input, significantly expanding its applicability. It achieves a statistically significant improvement in AUC compared to the current second-best model. Furthermore, MsgaBpred demonstrates strong generalizability, maintaining top performance across multiple independent datasets.

MsgaBpred integrates both ESM-C and ESM-IF1, two large pretrained protein language models, to extract evolutionary sequence embeddings and inverse folding representations. Notably, ESM-C outperforms ESM-2 with a similar number of parameters, offering more informative sequence representations for downstream tasks. The multi-scale GCN module effectively captures both short- and long-range dependencies by leveraging varying receptive fields, while the additive attention mechanism helps identify key residues by evaluating their global importance within the sequence context.

Despite its promising results, MsgaBpred has certain limitations. Conformational B-cell epitopes often depend on surface topology and dynamic structural rearrangements, but AlphaFold3 generates static 3D structures, and some epitope regions may have low confidence scores (e.g., low pLDDT), which can hinder accurate prediction. Additionally, while the multi-scale GCN and additive attention modules partially capture global context, their ability to fully model long-range interactions remain limited.

In future work, we plan to incorporate molecular dynamics simulations to better account for protein conformational flexibility and dynamic behavior, thereby complementing the static structural predictions provided by AlphaFold3. Furthermore, we intend to develop novel model architectures capable of capturing more extensive global context, thereby improving the prediction accuracy of conformational B-cell epitopes.

## Supporting information

S1 Table Table presents the performance of using different features on the independent test data. (DOCX)

S2 Table Table compares the performance of MsgaBpred with state-of-the-art methods on epitope3D dataset. (DOCX)

S3 Table Table presents the prediction results on the independent test set after excluding the respective module. (DOCX)

S4 Table Table provides a comprehensive comparison of the MsgaBpred model and state-of-the-art methods across multiple datasets. (DOCX)

S5 Table Table presents a comparison of our model’s performance when DSSP maintains its dimensionality unchanged and when it is mapped to a higher dimensionality. (DOCX)

S1 Fig Fig shows a positive correlation between GDT scores and AUPR values on epitope3D dataset. (DOCX)
