# Supplementary material for: MsgaBpred: A B-cell epitope predictor integrating AlphaFold3-predicted structures with multi-scale GCNs and pre-trained language model ESM-C
Source: PLoS Comput Biol. 2026 Apr 28;22(4):e1014195. doi: 10.1371/journal.pcbi.1014195 (PMC13123994; doi:10.1371/journal.pcbi.1014195)
Supplement: S1 Table — (DOCX) [file pcbi.1014195.s001.docx]

**S1 Table**. The performance of using different features on the independent test data.

| Feature group | AUC | AUPR | Pre | F1 | MCC | BACC |
| --- | --- | --- | --- | --- | --- | --- |
| DSSP | 0.622 | 0.111 | 0.114 | 0.189 | 0.100 | 0.589 |
| ESM-IF1 | 0.708 | 0.162 | 0.150 | 0.235 | 0.166 | 0.640 |
| ESM-C | 0.714 | 0.195 | 0.210 | 0.269 | 0.195 | 0.626 |
| ESM-IF1+DSSP | 0.711 | 0.176 | 0.165 | 0.240 | 0.163 | 0.623 |
| ESM-C+DSSP | 0.729 | 0.204 | **0.218** | 0.280 | 0.209 | 0.636 |
| ESM-C+ESM-IF1 | 0.725 | 0.202 | 0.209 | 0.266 | 0.193 | 0.626 |
| MsgaBpred(Ours) | **0.744** | **0.227** | **0.218** | **0.293** | **0.225** | **0.654** |
