# Supplementary material for: MsgaBpred: A B-cell epitope predictor integrating AlphaFold3-predicted structures with multi-scale GCNs and pre-trained language model ESM-C
Source: PLoS Comput Biol. 2026 Apr 28;22(4):e1014195. doi: 10.1371/journal.pcbi.1014195 (PMC13123994; doi:10.1371/journal.pcbi.1014195)
Supplement: S2 Table — (DOCX) [file pcbi.1014195.s002.docx]

**S2 Table**. Performance comparison of MsgaBpred with state-of-the-art methods on epitope3D dataset.

| Method | AUC | BACC | F1 | MCC |
| --- | --- | --- | --- | --- |
| SEPPA 3 | 0.52 | 0.52 | 0.14 | 0.02 |
| Disco Tope-2.0 | 0.49 | 0.5 | 0.11 | -0.01 |
| Ellipro | 0.44 | 0.44 | 0.11 | -0.06 |
| BepiPred-2.0 | 0.54 | 0.55 | 0.15 | 0.04 |
| epitope3D | 0.49 | 0.49 | 0.02 | -0.02 |
| BepiPred-3.0 | 0.71 | 0.57 | 0.19 | 0.08 |
| Disco Tope-3.0 | 0.71 | 0.57 | 0.2 | 0.09 |
| GraphBepi | 0.64 | 0.62 | 0.28 | 0.16 |
| EpiGraph | 0.73 | 0.62 | 0.29 | 0.19 |
| MsgaBpred(Ours) | **0.75** | **0.65** | **0.31** | **0.24** |
