# Supplementary material for: MsgaBpred: A B-cell epitope predictor integrating AlphaFold3-predicted structures with multi-scale GCNs and pre-trained language model ESM-C
Source: PLoS Comput Biol. 2026 Apr 28;22(4):e1014195. doi: 10.1371/journal.pcbi.1014195 (PMC13123994; doi:10.1371/journal.pcbi.1014195)
Supplement: S3 Table — (DOCX) [file pcbi.1014195.s003.docx]

**S3 Table**. The predictive performance on the independent test data when removing module.

| Module | AUC | AUPR | Pre | F1 | MCC | BACC |
| --- | --- | --- | --- | --- | --- | --- |
| w/o 1 MGCN | 0.730 | 0.215 | **0.232** | 0.282 | 0.211 | 0.629 |
| w/o 2 MGCN | 0.568 | 0.104 | 0.103 | 0.170 | 0.068 | 0.557 |
| w/o Attention | 0.733 | 0.212 | 0.224 | 0.285 | 0.215 | 0.639 |
| MsgaBpred(Ours) | **0.744** | **0.227** | 0.218 | **0.293** | **0.225** | **0.654** |

Note: w/o means without the corresponding module. MGCN means the muti-scale graph convolution module. Attention means the additive attention
