# Supplementary material for: MsgaBpred: A B-cell epitope predictor integrating AlphaFold3-predicted structures with multi-scale GCNs and pre-trained language model ESM-C
Source: PLoS Comput Biol. 2026 Apr 28;22(4):e1014195. doi: 10.1371/journal.pcbi.1014195 (PMC13123994; doi:10.1371/journal.pcbi.1014195)
Supplement: S4 Table — (DOCX) [file pcbi.1014195.s004.docx]

**S4 Table**. Performance comparison of MsgaBpred with state-of-the-art methods on external datasets.

| Methods | DiscoTope3_Foldx | DiscoTope3_Solved | DiscoTope3_Af2 |
| --- | --- | --- | --- |
| WUREN | 0.218 | 0.213 | 0.193 |
| PINet | 0.252 | 0.203 | 0.116 |
| PECAN | 0.162 | 0.163 | 0.161 |
| DiscoTope 3.0 | 0.221 | 0.223 | 0.232 |
| BepiPred 3.0 | 0.177 | 0.177 | 0.177 |
| Epitope3D | 0.181 | 0.177 | 0.169 |
| EPCES | 0.191 | 0.191 | 0.181 |
| EPSVR | 0.179 | 0.179 | 0.166 |
| ElliPro | 0.183 | 0.185 | 0.187 |
| MsgaBpred(Ours) | 0.368 | 0.362 | 0.329 |
