# Supplementary material for: MsgaBpred: A B-cell epitope predictor integrating AlphaFold3-predicted structures with multi-scale GCNs and pre-trained language model ESM-C
Source: PLoS Comput Biol. 2026 Apr 28;22(4):e1014195. doi: 10.1371/journal.pcbi.1014195 (PMC13123994; doi:10.1371/journal.pcbi.1014195)
Supplement: S5 Table — (DOCX) [file pcbi.1014195.s005.docx]

**S5 Table**. Performance comparison of MsgaBpred with our model, which keeping DSSP its ordinary 13 dimension.

| Model | AUC | AUPR | Pre | F1 | MCC | BACC |
| --- | --- | --- | --- | --- | --- | --- |
| MsgaBpred(13) | 0.740 | 0.214 | 0.217 | 0.281 | 0.210 | 0.637 |
| MsgaBpred(ours) | **0.744** | **0.227** | **0.218** | **0.293** | **0.225** | **0.654** |
