# Supplementary material for: MsgaBpred: A B-cell epitope predictor integrating AlphaFold3-predicted structures with multi-scale GCNs and pre-trained language model ESM-C
Source: PLoS Comput Biol. 2026 Apr 28;22(4):e1014195. doi: 10.1371/journal.pcbi.1014195 (PMC13123994; doi:10.1371/journal.pcbi.1014195)
Supplement: S1 Fig — (DOCX) [file pcbi.1014195.s006.docx]

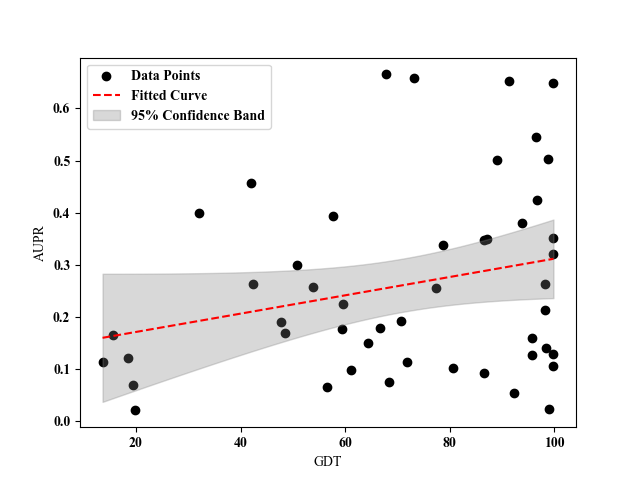


**S1 Fig.**The positive correlation between the predicted quality of AlphaFold3 as measured by GDT and the performance of MsgaBpred on the epitope3D test set. Each protein’s corresponding GDT and AUPR values are denoted by black scatter points, and a red line represents the GDT and AUC for functional relationships in graph.
